# Skin prick test assessment of sensitization to profilin in pollen allergic patients in the population of south-west Poland

**DOI:** 10.1186/1939-4551-8-S1-A197

**Published:** 2015-04-08

**Authors:** Anna Kosowska, Marek Jutel, Joanna Przezdziecka-Dolyk

**Affiliations:** 1 Wroclaw Medical University, Department of Clinical Immunology, Poland; 2 All-Med Medical Research Institute, Poland

## Background

Panallergens (profilins, polcalcins and non-specific lipid transfer proteins) are responsible for polysensitization to various allergens including pollen and food allergens in a number of individuals which might account for a problem in distinguishing between true allergy from cross reactivity in allergy testing.

A broad range of pollen- and plant-derived allergens contain profilins and it has been shown in selected populations that 10% to 20% of patients with pollen allergy are sensitized to them. Patients with profilin-specific IgE antibodies in serum show higher risk of developing of the sensitization to multiple allergens.

## Methods

Sensitization to common pollen allergens using commercial extracts (Allergopharma, Germany) and profilin (provided by ALK) was assessed in 77 patients suffering from seasonal allergic rhinitis during the pollen season (37 men, 40 women, age 18-76 years) by skin prick tests (SPT). The wheal mean diameter of > 3 mm was considered positive.

## Results

18,18% of patients showed positive test results to profilin. 62,33% of patients had positive SPT to grass pollen and 57,14% were sensitized to mugwort pollen. Profilin positive subjects were sensitized to various pollen allergens and in some cases also to fungal allergens, but those patients were most frequently sensitized to mugwort, birch and plantain pollen (p<0,05). In both, birch or mugwort pollen positive patients sensitization to profilin was more prevalent than in the birch or mugwort negative subjects (both p<0,05).

## Conclusions

Sensitization to profilin is prevalent among pollen allergic population in South-West Poland. Prevalence of sensitization to profilin can differ in patients allergic to mugwort, birch or plantain. Profilin sensitization is a risk factor for allergic reactions to multiple pollen and food allergen sources. Patients sensitized to panallergens should be tested by an adequate panel of allergenic molecules in order to identify the allergens that are responsible for the allergic disease.

